# Effect of LYRM1 knockdown on proliferation, apoptosis, differentiation and mitochondrial function in the P19 cell model of cardiac differentiation in vitro

**DOI:** 10.1007/s10863-015-9638-4

**Published:** 2016-01-13

**Authors:** Yu-Mei Chen, Xing Li, Gui-Xian Song, Ming Liu, Yi Fan, Li-Jie Wu, Hua Li, Qi-Jun Zhang, Yao-Qiu Liu, Ling-Mei Qian

**Affiliations:** Department of Cardiology, The First Affiliated Hospital of Nanjing Medical University, No. 300 Guangzhou Road, Nanjing, 210029 Jiangsu Province People’s Republic of China; Department of MICU, Nanjing Maternity and Child Health Care Hospital Affiliated to Nanjing Medical University, Nanjing, 210029 China

**Keywords:** Congenital heart disease, LYRM1, P19 cells, Mitochondria, RNA interference

## Abstract

To explore the effects of LYRM1 knockdown on proliferation, apoptosis, differentiation and mitochondrial function in the embryonic carcinoma (P19) cell model of cardiac differentiation. Knockdown of LYRM1 using small interfering RNA (siRNA) was confirmed by quantitative real-time PCR. Cell Counting Kit-8(CCK-8) proliferation assays and cell cycle analysis demonstrated that LYRM1 gene silencing significantly inhibited P19 cell proliferation. Flow cytometry and measurement of their caspase-3 activities revealed that knockdown of LYRM1 increased P19 cell apoptosis. Observation of morphological changes using an inverted microscope and expression analysis of specific differentiation marker genes using quantitative real-time PCR and Western blotting revealed that knockdown of LYRM1 significantly inhibited the differentiation of P19 cells into cardiomyocytes. Furthermore, real-time quantitative PCR applied to detect mitochondrial DNA (mtDNA) copy number implied that there was no significant difference in the LYRM1 knockdown group compared with the control group. Cellular ATP production investigated by luciferase-based luminescence assay was dramatically decreased in differentiated cells transfected with LYRM1 RNAi. Fluorescence microscopy and flow cytometery were used to detect the reactive oxygen species (ROS) and the mitochondrial membrane potential (MMP) showed that the level of ROS was dramatically increased and MMP was obviously decreased in differentiated cells transfected with LYRM1 RNAi. Collectively, knockdown of LYRM1 promoted apoptosis and suppressed proliferation and differentiation in P19 cells. In addition, knockdown of LYRM1 induced mitochondrial impairment in P19 cells during differentiation, which was reflected by decreased ATP synthesis, lower MMP and increased ROS levels.

## Introduction

Heart development is an extremely complex process, which involves precisely temporal and spatial expression of many genes associated with embryonic development (Christoffels et al. 2000). Research shows cardiac structure and function are quite sensitive to genetic perturbation during cardiogenesis in human and that mutations in components of the cardiac gene network can lead to adverse consequences in the form of congenital heart disease (CHD) (Olson 2006). At present, congenital heart disease, broadly grouped into two major categories: morphological and functional abnormalities of the heart and great vessels, is the most common kind of congenital abnormalities found at birth and the significant cause of neonatal morbidity and mortality, which brings a heavy burden on families and society (Hoffman et al. 2004). Thus, exploring molecular etiology is still the key to the prevention and treatment of CHDs.

In our earlier studies, we found and cloned *Homo sapiens* LYR motif containing 1 (LYRM1), a novel gene that showed the highest expression level in adipose tissue and also abundantly expressed in human heart tissue (Qiu et al. 2009). We found that LYRM1 had an effect on differentiation, proliferation and apoptosis in 3 T3-L1 pre-adipocytes and also influenced mitochondrial function in mature 3 T3-L1 adipocytes (Cao et al. 2010; Qiu et al. 2009; Zhu et al. 2012). In addition, Studies have shown that LYRM1 was a member of the Complex1_LYR superfamily, and LYR proteins were predominantly mitochondrial proteins and influenced mitochondrial homeostasis (Angerer 2013; Pagliarini et al. 2008). These findings indicate that LYRM1 plays a significant role in cell growth, apoptosis and mitochondrial function. Further studies by our group revealed that overexpression of LYRM1 promoted proliferation and inhibited apoptosis in embryonic myocardial cells, which Indicated that the LYRM1 gene may play a vital role in the development of the human heart (Zhu et al. 2010). However, the specific mechanism of LYRM1 in heart development remains unclear.

P19 cells, isolated from an experimental embryo-derived mouse teratocarcinoma, are used widely as a suitable myocardial cell model to investigate cardiac differentiation at the molecular and functional levels (van der Heyden et al. 2003; Wen et al. 2007). Accordingly, we chose this model to investigate the effect of LYRM1 knockdown on cell proliferation, apoptosis, differentiation and mitochondrial function.

## Materials and methods

### Cell culture and differentiation

P19 cells were purchased from the American Type Culture Collection (ATCC, Manassas, VA, USA). P19 cells were cultured in complete medium composed by α-MEM culture medium (Gibco, NY, USA) with 10 % fetal bovine serum (FBS, Gibco BRL), 100μg/ml penicillin, and 100 μg/ml streptomycin at 37 °C in 5 % CO2. To induce cardiac differentiation, P19 cells were maintained in complete medium supplemented with 1 % dimethyl sulfoxide (DMSO, Sigma, St. Louis, MO, USA) and aggregated over the following 4 days to form embryoid bodies (EBs), and the medium was replenished every 24 h. The formed EBs were transferred to 6-well culture plates and cultured in complete medium free of DMSO for an additional 4 or 6 days. The morphologic changes in P19 cells were observed and photographed using an inverted microscope (Nikon, Japan). Cell total RNAs and proteins were extracted on day 0, 6 and 10 during differentiation.

### Construction of RNAi lentiviral vector and establishment of LYRM1 silenced cells

RNAi lentiviral vector was constructed as previously described (Zhu et al. 2012). Briefly, the designed short hairpin RNA (shRNA) construct contained a 19-nt double-stranded LYRM1 sequence, which was an inverted complementary repeat that formed a loop sequence (5′-CTCGAG- 3′). The 5′overhangs, CCGG and AATT, were ligated into AgeI- and EcoRI-digested pgLV-U6-puro lentivirus vector in the positive-sense and antisense strands, respectively. The recombinant vector was named pgLV-U6-puro-LYRM1-shRNA. The negative control vector (pgLV-U6-puro-NC-shRNA) contained a nonsense shRNA insert to control any effects caused by non-RNAi mechanisms. The sequences of the cDNA fragment (positive-sense strand) were as follows: LYRM1, 5′GCAATCATTTCTAGACTAA; negative control, 5′TTCTCCGAACGTGTCACGT.

We co-transfected the HEK-293 T cells with three optimized packaging plasmids (pGag/Pol, pRev and pVSV–G) and the pgLV-U6-puro-LYRM1-shRNA or pgLV-U6-puro-NC-shRNA expression clone construct, which could produce lentiviral stocks with a suitable titer. The collected lentiviral supernatant filtered through a 0.45 um membrane were supplemented with hexadimethrine bromide (polybrene) to a final concentration of 5μg/ml and applied directly to P19 cells. Stably transduced P19 cells were selected in medium containing 2 μg/ml puromycin (Sigma-Aldrich, St. Louis, MO, USA) over 2 weeks. The efficiency of knockdown was detected by real-time quantitative polymerase chain reaction (qPCR) using the primers shown in Table 1.

**Table 1:** PCR primer sequences used

Gene	Forward primer (5′-3′)	Reverse primer (3′-5′)
GAPDH	ATTCAACGGCACAGTCAA	CTCGCTCCTGGAAGATGG
GATA4	ACGGAAGCCCAAGAACCTG	GCTGCTGTGCCCATAGTGAG
NKX2.5	GACAGCGGCAGGACCAGACT	GCCATAGGCATTGAGACCCAC
CYTB	TTTTATCTGCATCTGAGTTTAATCCTGT	CCACTTCATCTTACCATTTATTATCGC
28S	GGCGGCCAAGCGTTCATAG	AGGCGTTCAGTCATAATCCCACAG
LYRM1	AGGGCAGATGGAAGACACC	GATGGATAGGGCGTGGATAA

### Cell growth assay

Cell proliferation rate was evaluated using the WST-8 Cell Counting Kit-8 (Dojindo, Tokyo, Japan) according to the manufacturer’s instructions. Briefly, seeded in 96-well plate, the cells were incubated in a-MEM supplemented with 10 % FBS for 4 days. CCK-8 reagent (10 μl, 1 mg/ml) was added and incubated for 3 h at 37 °C. Then, we used a microplate reader (Bio-Rad, Hercules, CA, USA) to measure the absorbance at 450 nm.

### Cell cycle assay

In order to synchronize the cell cycle, we cultivated the cells in serum-free α-MEM for 24 h before analysis. After serum deprivation, Cells were incubated with complete medium for 0 and 24 h. Following incubation, cells were harvested using trypsin/EDTA, fixed in 70 % ethanol at 4 °C overnight, and cellular DNA was stained with 500 μL propidium iodide (PI) (BD Bioscience, San Diego, CA, USA). The DNA content of 104 cells was analyzed by a FACSCalibur flow cytometer (BD Bioscience) and the population in each cell cycle phase was analyzed using FlowJo software (FlowJo, Ashland, OR, USA).

### Apoptosis assay

Apoptotic cells were assayed by flow cytometric analysis of annexin V–allophycocyanine (APC) and 7-amino-actinomycin D (7-AAD) staining. The cells were cultivated in serum-free α-MEM for 24 h to induce apoptosis, collected by trypsin without EDTA (Gibco BRL, USA) and washed with phosphate-buffered saline (PBS, Gibco BRL). After centrifuged and resuspended, the cells were stained with 5 μL Annexin V-APC and 5 μL 7-AAD at room temperature for 15 min (Biovision, CA, USA). Flow cytometry was then used to analyze the cells immediately.

### Assay of caspase-3 activity

We used a CaspGLOW Fluorescein Active Caspase-3 Staining Kit (Biovision, CA, USA) to analyze the caspase-3 activity of cells. Cells (1 × 106/ml) were induced apoptosis by method of serum deprivation and we concurrently incubated a control culture without induction. An additional negative control can be prepared by adding the caspase inhibitor Z-VAD-FMK at 1 μl/ml to an induced culture to inhibit caspase-3 activation. We put aliquot 300 μl each of the induced and control cultures into eppendorf tubes and added 1 μl of FITC-DEVD-FMK into each tube and incubated for 0.5–1 h at 37 °C incubator with 5 % CO2. Then, we centrifuged cells at 3000 rpm for 5 min and resuspended cells in 0.5 ml of Wash Buffer, and centrifuged again, resuspended cells in 300 μl of Wash buffer. The samples were analyzed by flow cytometry using the FL-1 channel (BD Bioscience).

### Quantitative real-time PCR

Total RNAs from P19 cells transfected with lentiviral-LYRM1-shRNA or lentiviral-NC-shRNA were extracted using TRIzol reagent (Invitrogen). Reverse transcription was performed using a RevertAid First Strand cDNA Synthesis Kit (Thermo Scientific, Pittsburgh, PA, USA). Real-time PCR (SYBR Green method) was performed using 7500 Fast System Real-Time PCR cycler (Applied Biosystems) according to the manufacturer’s protocols. The relative gene expression levels were quantified based on the Ct and normalized against a reference gene, GAPDH. The sequences of the primers are shown in Table 1.

Real-time PCR was used to determine the relative amounts of mitochondrial DNA (mtDNA), as described previously (Kaaman et al. 2007). We exacted DNA from differentiated cells using a DNA Exaction Kit (QIAGEN, Dusseldorf, Germany). Two primer sets were used for PCR analysis. A 110-nt mtDNA fragment within the cytochrome B gene (CYTB) was used for the quantification of mtDNA. Previously, the PCR product had been inserted into the plasmid pMD-T18 and verified by DNA sequencing. According to the plasmid standards of known copy number,we got a log-linear standard curve, through which the CYTB copy numbers of the samples could be detected by real-time PCR conducted on the ABI 7500 Sequence Detection System. The 291-bp region of the nuclear gene for 28 S was used as normalization standard. The ratio of mtDNA to nuclear DNA was counted to reflect the copy number of mitochondrial per cell.

### Antibodies and Western blotting

Anti-GAPDH antibodies purchased from Proteintech Group (Proteintech, USA), anti-GATA4 and anti-Nkx 2.5 antibodies were both from Abcam (Abcam plc, Cambridge, UK). Total proteins from cultured cells were extracted using the Total Protein Extraction Kit (KeyGen, inc., China). Subsequently, the protein concentrations were measured by a bicinchoninic acid (BCA) assay kit (KeyGen, Nanjing, China). We separated the proteins by 10 % SDS–polyacrylamide gel electrophoresis, and then transferred onto polyvinylidene difluoride membranes. After blocked by the 5 % dried skimmed milk at room temperature for 2 h, the membranes were incubated with the corresponding primary antibodies overnight at 4 °C. We washed the membranes three times with Tris-buffered saline with Tween 20 (TBST), and used appropriate horseradish peroxidase–conjugated secondary antibody to incubate them at room temperature for 2 h. Proteins were detected using ECL plus reagents (Beyotime, Shanghai, China).

### Measurement of cellular ATP production

Using a Luciferase-Based Luminescence Assay Kit (Biyutian, Nantong, China), we measured the cellular adenosine triphosphate (ATP) on the 10th day of differentiation. Briefly, the differentiated P19 cells were homogenized in an ice-cold ATP-releasing buffer, and then we determined the ATP concentrations with a single-tube luminometer (Turner Biosystems, USA). The production of ATP was normalized to protein concentration determined by the BCA method.

### Fluorescence microscopy and flow cytometry

We measured the intracellular ROS levels by a 2′, 7′-dichlorodihydrofluorescein diacetate acetyl ester (H2-DCFDA) probe (Sigma, St. Louis, MO, USA) (Maxwell et al. 1999), and evaluated the mitochondria membrane potential (MMP) by a JC-1 fluorescent probe from the Mitochondrial Membrane Potential Detection Kit (Biyutian, China). The cells were incubated with 1 ml of JC-1 or 5 μM of DCFDA for 30 min at 37 °C, washed three times with pre-warmed PBS, and then imaged under a fluorescence microscopy. For flow cytometry, following trypsinization and centrifugation, the stained cells were resuspended in 300 μl PBS buffer. The fluorescence were analyzed with a FACScan flow cytometer with the CellQuest software (BD Biosciences, San Joes,CA, USA).

### Statistical analysis

Each experiment was performed at least three times. All data were expressed as means ±standard error of the mean (SED). Statistical analysis was performed using Student’s t-test with the SPSS 16.0 statistical software package (SPSS Inc., Chicago, IL, USA). The threshold of statistical significance was defined as *P* < 0.05.

## Results

### Appraisal of LYRM1 suppression efficiency

**Fig. 1:**
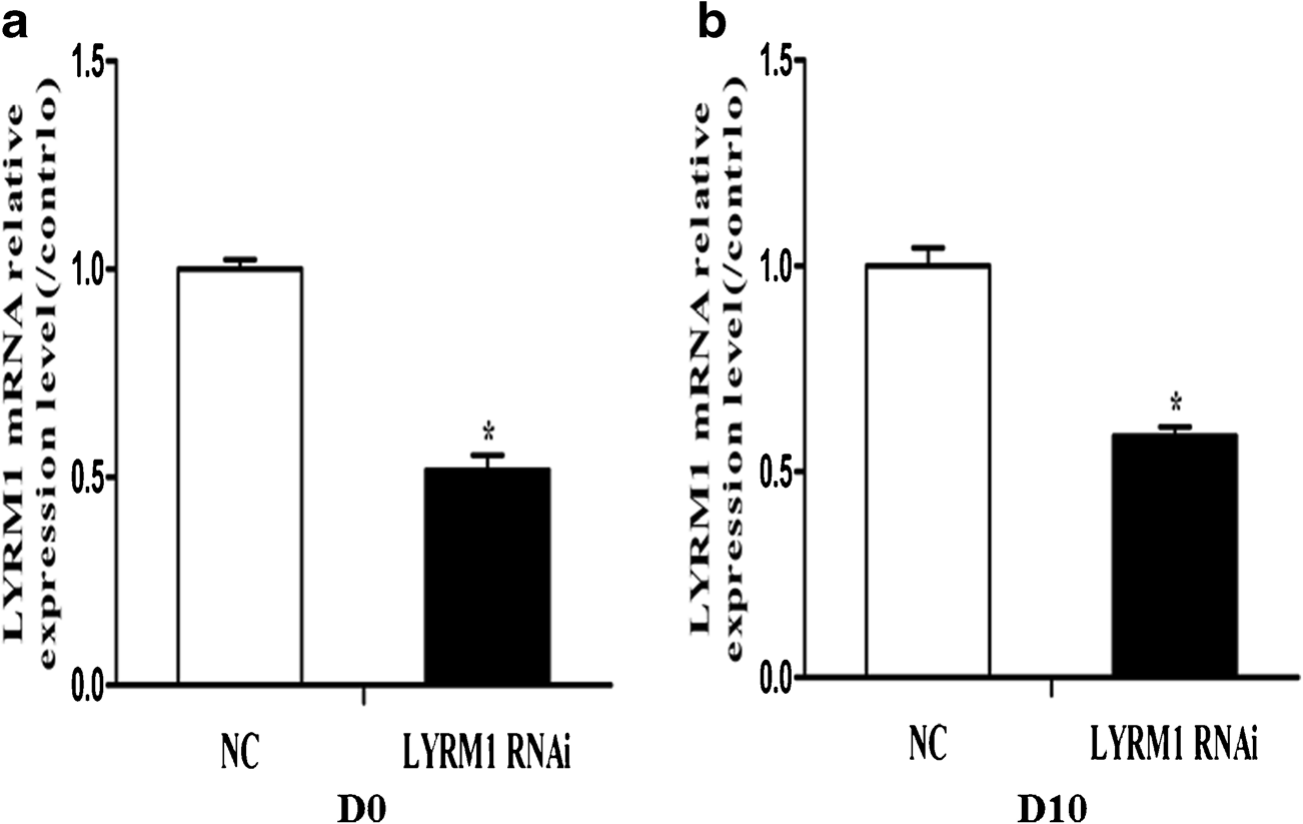
Appraisal of LYRM1 suppression efficiency. LYRM1 mRNA relative to GAPDH in the lentiviral-LYRM1-shRNA-transfected P19 cells was examined by real-time PCR in comparison with lentiviral-NC-shRNA-transfected P19 cells. The mRNA inhibitory efficiency at day 0 and 10 were 51 % and 58 %, respectively (*P < 0.05 vs. negative control (NC) cells)

The knockdown efficiency of LYRM1 was evaluated by real-time PCR. The relative LYRM1 mRNA level in P19 cells was normalized against the mRNA levels of a reference gene, GAPDH. As shown in Fig. 1, cells infected with pGLV–LYRM1–shRNA expressed significantly lower levels of LYRM1 mRNA than the cells transfected with the negative control (NC) lentiviral construct (*P < 0.05). The steady state LYRM1 mRNA level of the pGLV–LYRM1–shRNA-infected cells was 51 % that of the negative control cells before differentiation. On the 10th day of differentiation, the LYRM1 mRNA level of the pGLV–LYRM1–shRNA-infected cells was 58 % that of the negative control cells.

### LYRM1 gene silencing reduces P19 cell proliferation

**Fig. 2:**
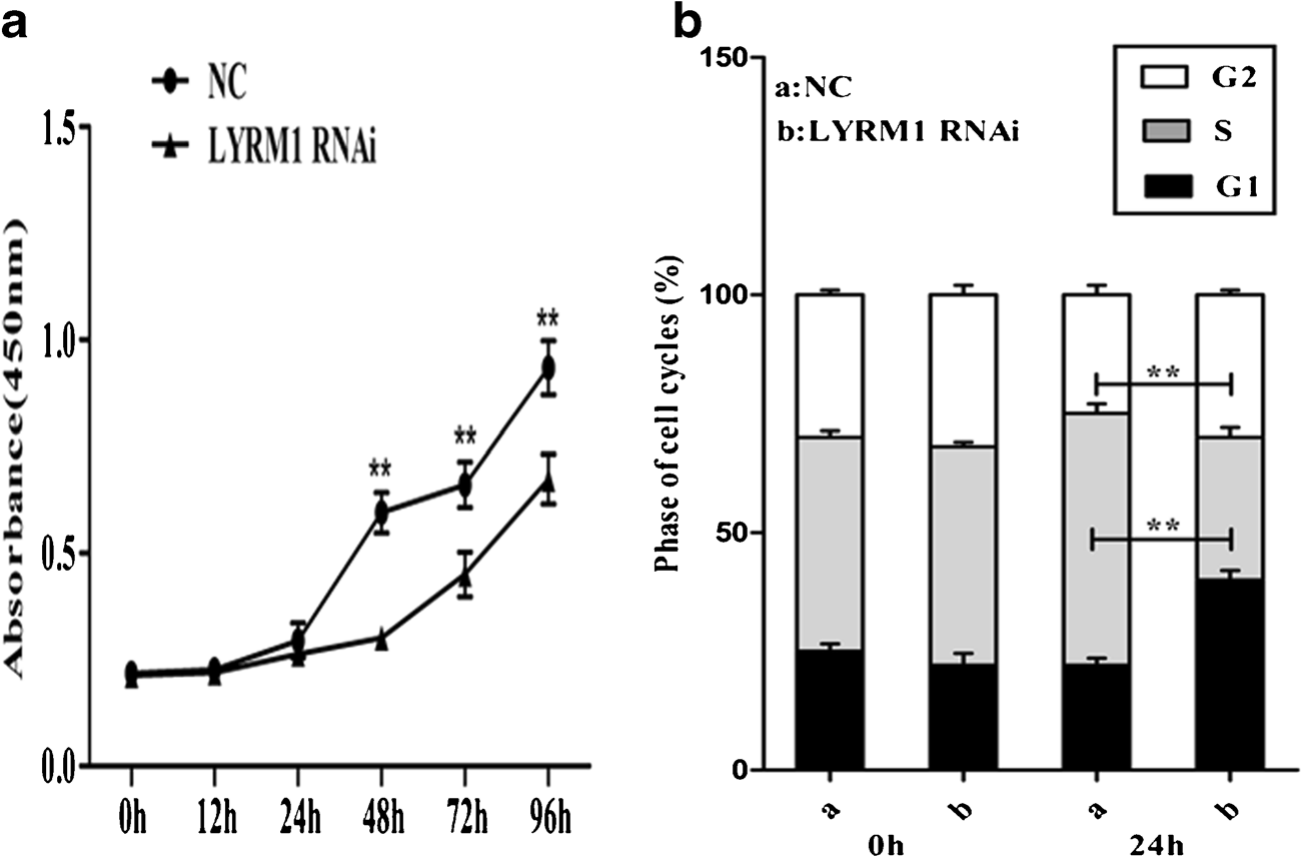
LYRM1 gene silencing reduces P19 cell proliferation. P19 cells were transfected with pGLV–LYRM1–shRNA (LYRM1 RNAi) or pGLV–NC–shRNA lentivirus (NC). Cell growth was quantified using the CCK-8 cell viability assay (**a**) and cell cycle analysis (**b**). Values are the mean ± SD of three independent experiments (*n* = 6,**P < 0.01)

We used the CCK-8 assay to assess the growth of LYRM1-silenced and NC P19 cells for 96 h. As shown in Fig. 2a, LYRM1-silenced cells significantly reduced growth rate compared to NC control cells. From 48 h onwards, the OD values of LYRM1-silenced cells were significantly lower than control cells (**P < 0.01). Further, we performed flow cytometry analysis of the cell cycle distribution in LYRM1-silenced and NC control P19 cells, and observed that the proportion of S phase cells in LYRM1-silenced P19 cells was much lower than NC control cells and G1 phase fraction in LYRM1-silenced P19 cells was distinctly increased (Fig. 2b).

### LYRM1 gene silencing induces P19 cell apoptosis

**Fig. 3:**
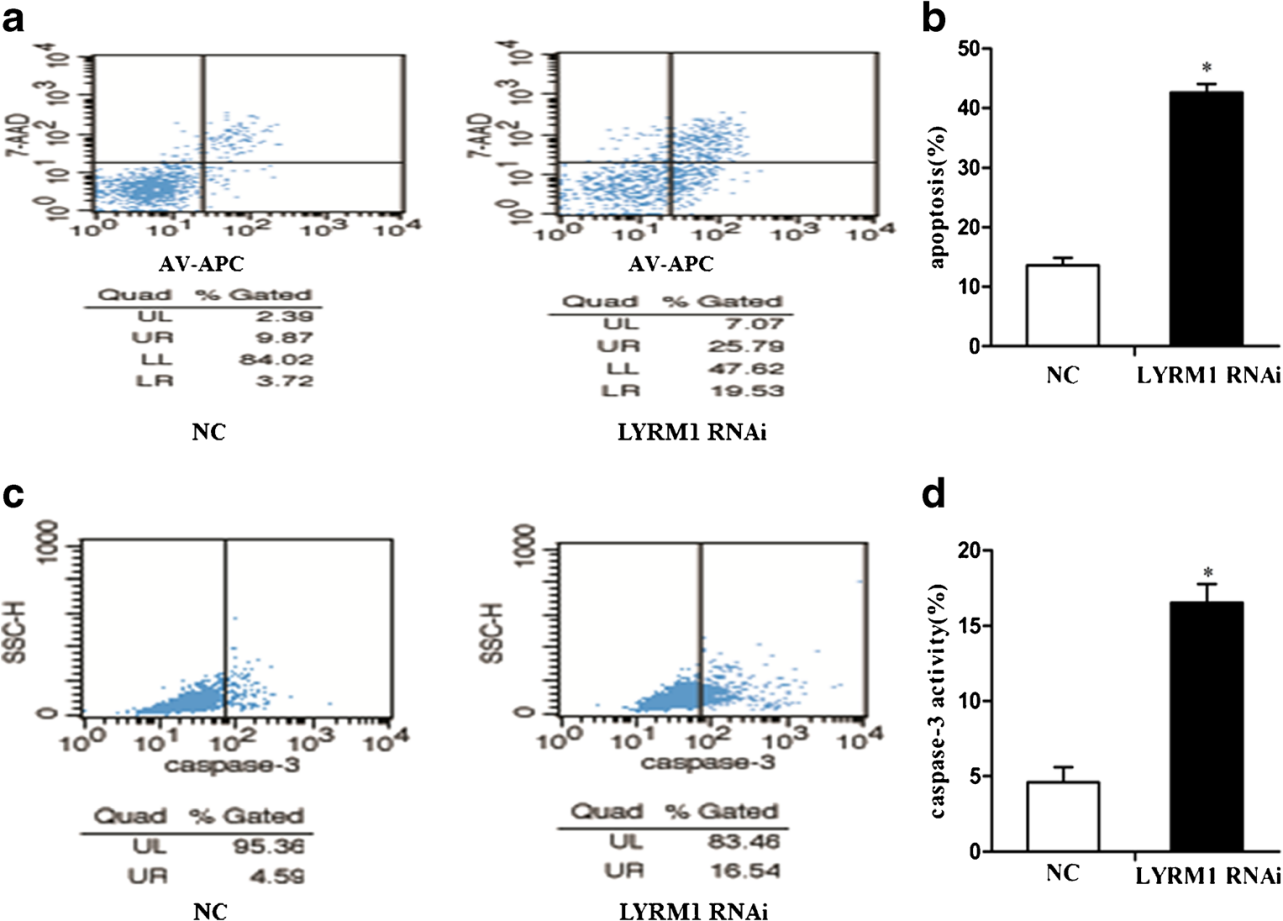
LYRM1 gene silencing induces P19 cell apoptosis in response to serum starvation. P19 cells were transfected with pGLV–LYRM1– shRNA (LYRM1 RNAi) or pGLV–NC–shRNA lentivirus (NC), serum starved for 24 h, and then cultured in complete media for 24 h. Apoptosis was quantified by flow cytometry using Annexin V-APC/7-AAD staining (**a**, **b**) and the caspase-3 activity assay (**c**,**d**). Values are the mean ± standard deviation of three independent experiments (*n* = 6,*P < 0.05)

To test the effect of LYRM1 knockdown on cell apoptosis, we induced apoptosis of P19 cells by serum starvation for 24 h, and then the cells were cultured in complete media for 24 h. After staining with Annexin V-APC/7-AAD probe, the numbers of apoptotic cells were quantified by flow cytometric analysis. The data indicated that LYRM1 knockdown increased the number of apoptotic cells in response to serum deprivation (*P < 0.05; Fig. 3a,b). In addition, the caspase-3 activity assay also demonstrated that LYRM1 knockdown increased the number of apoptotic cells in response to serum deprivation(*P < 0.05,Fig. 3c,d). These results indicated that LYRM1 gene silencing promotes serum deprivation-induced apoptosis.

### Differentiation of P19 cells cardiomyocytes

**Fig. 4:**
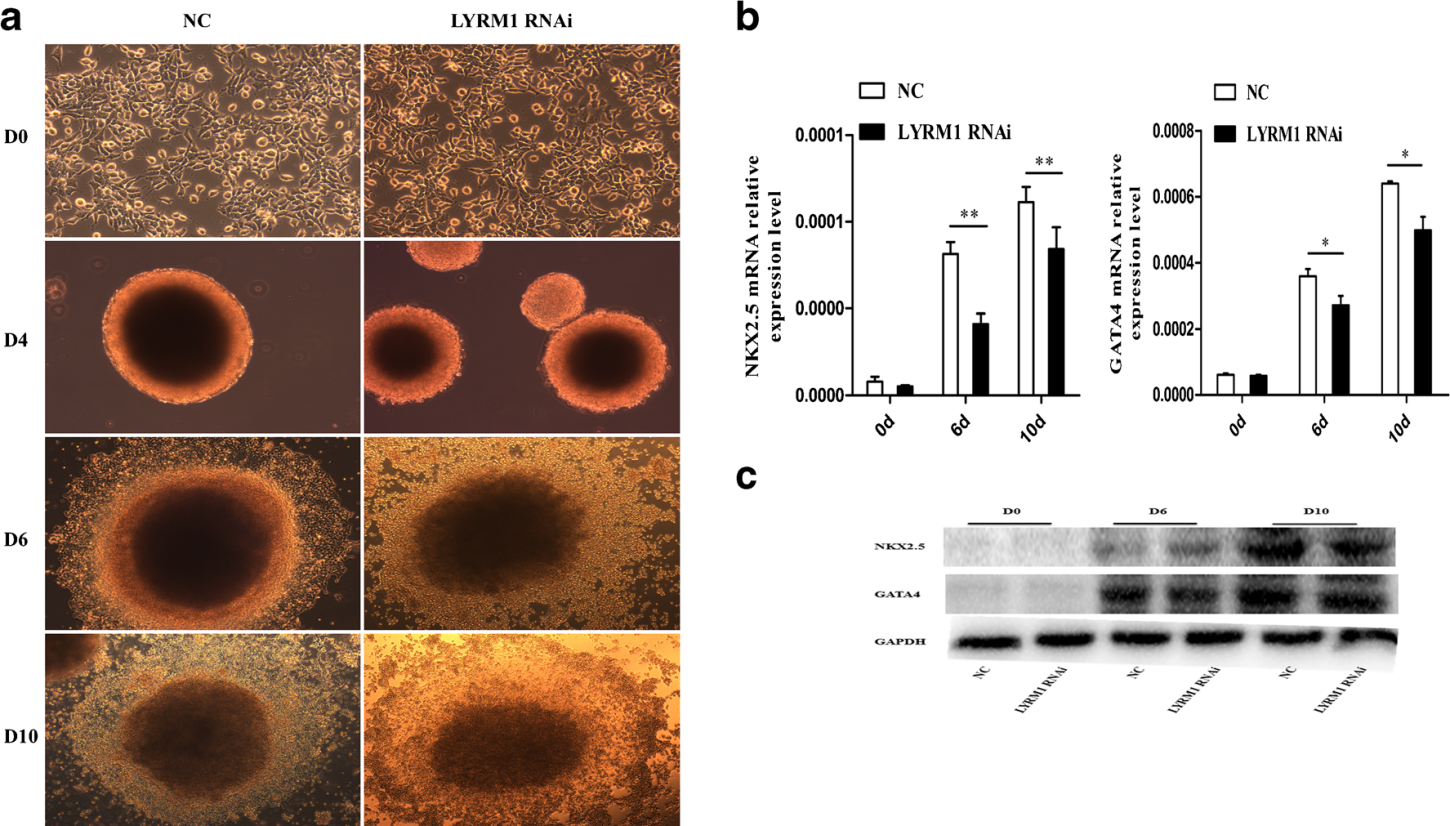
Effect of LYRM1 gene silencing on P19 cell differentiation. **a** P19 cell differentiation induced by 1 % DMSO. Micrographs show the morphological characteristics at day (D) 0, 4, 6, and 10 (×20). **b** qRT-PCR analysis of relative GATA4 and Nkx2.5 mRNA expression during differentiation. There were remarkable differences in the expression levels of these marker genes between the LYRM1 gene silencing and negative control groups. Data are the mean ± SD of three experiments(*P < 0.05,**p < 0.01). **c** Western blot detection of the protein levels of the marker genes during differentiation

P19 cells were differentiated into the cardiomyocytes by 1%DMSO. The morphological changes during differentiation were observed by an inverted microscope. As shown in Fig. 4a, P19 cells aggregated into embryoid bodies (EBs) in suspension from day 0 to day 4. At day 8, spontaneous beating was visible around the EBs. The beating cells continued to increase till day 10. Compared with the control group, the edge of EBs was not neat and smooth in the LYRM1 knockdown group. In the subsequent study, we detected the expression of cardiomyogenesis-specific markers (GATA4 and Nkx2-5) by real-time PCR,which shown that GATA4 and Nkx2-5 expression was significantly down-regulated during P19 cell differentiation into cardiomyocytes on days 6 and 10 in the LYRM1 knockdown group compared with the control group at the same time points (Fig. 4b,c; *P<0.05, **p < 0.01). Although the expression of cardiomyogenesis-specific markers detected by Western blots had no marked difference during P19 cell differentiation into cardiomyocytes on days 6, there was significantly down-regulated on days 10 in the LYRM1 knockdown group compared with the control group. These results suggested that LYRM1 knockdown suppressed DMSO-induced myocardial cell differentiation of P19 cells.

### Effect of LYRM1 on mitochondrial DNA copy number

**Fig. 5:**
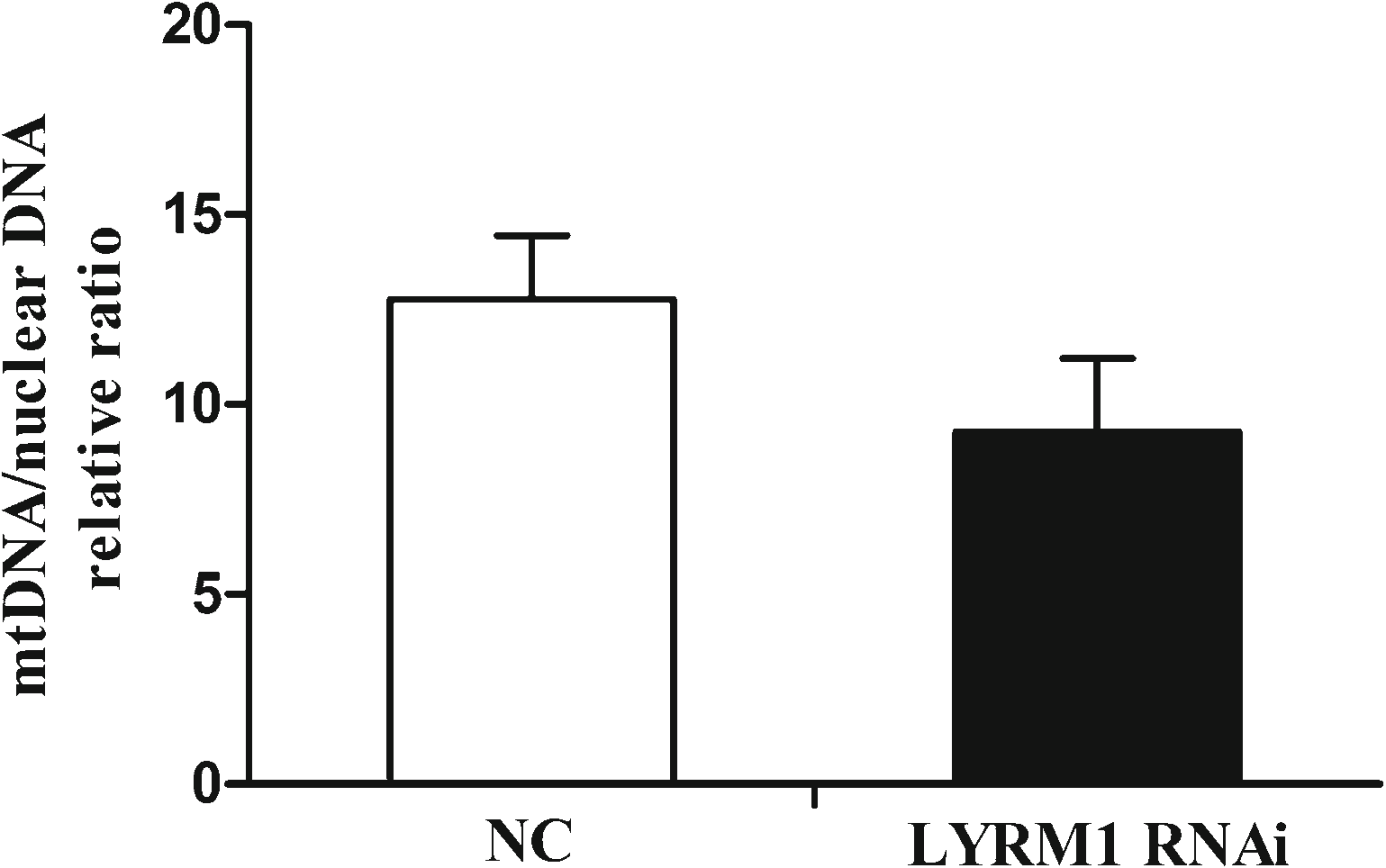
Effects of LYRM1 knockdown on the mitochondrial DNA copy number in differentiated cells. P19 cells transfected with pGLV–LYRM1– shRNA and the negative control(NC) cells were induced to differentiate. On the 10th day of differentiation, cellular mtDNA content was evaluated by real-time PCR analysis with primers designed to the CytB and 28S rRNA genes (*n* = 6). The CytB to28S rRNA gene ratio reflects the concentration of mitochondria per cell. *P* > 0.05 in comparison with negative control (NC) cells

The mtDNA copy number per mitochondrion is regarded generally as constant in most mammalian cell types(Robin and Wong 1988). Therefore, the mtDNA copy number is generally considered as the relative quantity of mitochondria and reflects mitochondrial function. We used real-time PCR to determine the mtDNA copy number in LYRM1 silenced and control cells and the results showed that there was no marked difference in mtDNA copy number between the two groups (Fig. 5,*P* > 0.05).

### Cellular ATP production was decreased upon LYRM1 knockdown

**Fig. 6:**
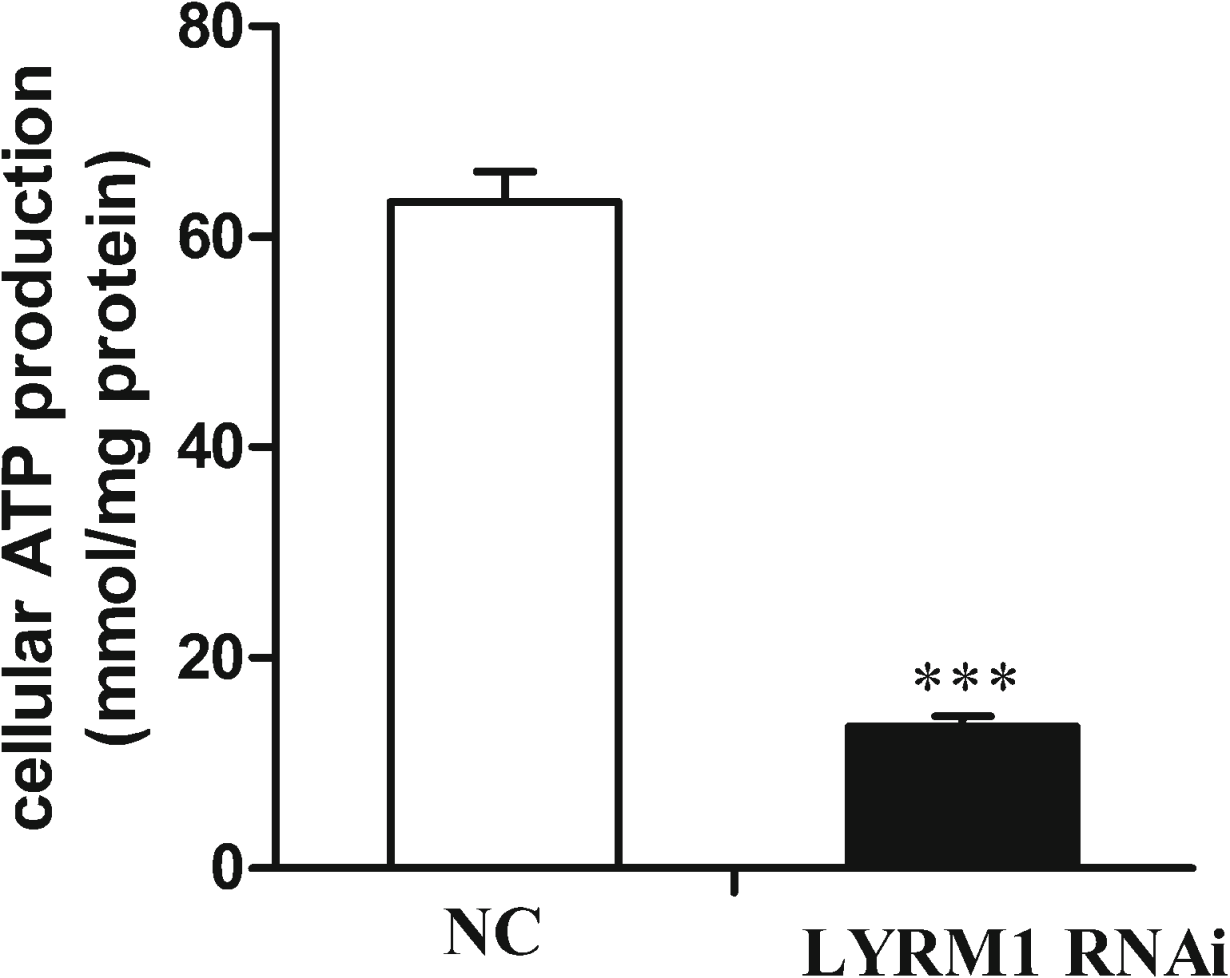
Effects of LYRM1 knockdown on intracellular ATP production in the differentiated cells. On the 10th day of differentiation, total cellular ATP levels were measured and normalized to the protein concentration. The results are representative of those values obtained from three independent experiments (*n* = 6). ***P < 0.001 in comparison with negative control (NC) cells

In cardiomyocytes, energy mainly originates from mitochondria where ATP is generated. Impaired mitochondria may lead to a lack of ATP. Here, we found that the cellular ATP production decreased dramatically in the LYRM1 silenced cells (Fig. 6, *** *P* < 0.001).

### Effects of LYRM1 knockdown on intracellular ROS levels and MMP

**Fig. 7:**
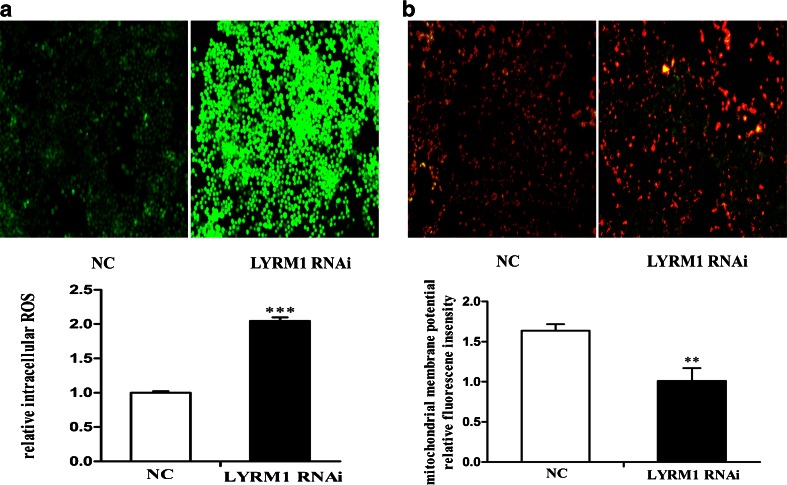
Effects of LYRM1 knockdown on intracellular reactive oxygen species (ROS) content and mitochondrial membrane potential (MMP) in the differentiated cells. (A)On the 10th day of differentiation, ROS levels were detected using a fluorescence microscopy (exCitation at 488 nm, emission at 530 nm) and fluorescence activated cell sorting (FACS) analysis. (*n* = 6, ***P < 0.001 vs. negative control (NC) cells). (B)On the 10th day of differentiation, MMP was detected by a fluorescence microscopy and FACS. (*n* = 6, **P < 0.01 vs. the NC cells)

In this study, we measured ROS levels by fluorescence microscopy and flow cytometry following treatment of cells with the fluorescent H2-DCFDA probe. As shown in Fig. 7, the fluorescence signals in LYRM1-silenced cells were higher than those of the control, indicating that silencing LYRM1 increased ROS generation (***P < 0.001). JC-1 is a fluorescent probe that applied widely to assess the MMP. In the mitochondrial matrix, JC-1 aggregates into a polymer to generate a red fluorescence at the high level of MMP, while JC-1 produces a green fluorescence without aggregating at the low level of MMP. In this study, fluorescence microscopy and flow cytometry were used to evaluate MMP reflected by the relative proportion of red and green fluorescence. Both methods showed that silencing LYRM1 produced a decrease in the MMP (Fig.7, ** *P* < 0.01).

## Discussion

The majority of scientists believed that CHD arises through various combinations of genetic and environmental contributors (Blue et al. 2012; Nora 1968). Although many genes have been found in the cardiogenesis process through animal studies, only a few genes related to CHD in humans have been identified (Joziasse et al. 2008). To date, the precise molecular mechanisms of CHD are still unclear.

In our previous trials, multi-tissue expression analysis of LYRM1 supported a role for LYRM1 in human heart development (Qiu et al. 2009). In addition, we found that LYRM1 promoted proliferation and inhibited apoptosis in embryonic myocardial cells, which might have the potential to modulate cell growth, apoptosis, and heart development(Zhu et al. 2010). Therefore, we aim to interpret the effect of LYRM1 on heart development by silencing its expression using small interfering RNA (siRNA).

CHD is strongly linked with the differentiation, proliferation, and apoptosis of myocardial cells (Levy et al. 2007a; Levy et al. 2007b). Therefore, we investigated the effects of LYRM1 knockdown on P19 cell differentiation, proliferation, and apoptosis by construction of the stable LYRM1 silenced cell line. As shown by the morphological changes and expression of cardiomyogenesis-specific molecular markers (GATA4 and Nkx2.5), we observed that knockdown of LYRM1 inhibited the differentiation of P19 cells into cardiomyocytes. Furthermore, the CCK-8 assay showed that P19 cells silencing LYRM1 grew slower than control cells, and cell cycle analysis indicated a remarkable decrease in the percentage of cells entering the S-phase and distinctly increased G1 phase fraction in LYRM1-silenced P19 cells. Meanwhile, Flow cytometry and measurement of caspase-3 activity revealed that apoptosis of P19 cells with silencing LYRM1 was significantly upregulated. These data indicated that LYRM1 knockdown dramatically inhibited proliferation, differentiation and promoted apoptosis in P19 cells.

Embryonic fetal heart growth depends on the balance between myocyte proliferation and apoptosis, and inadequate proliferation or excess apoptosis can directly or indirectly result in CHD (Fiorina et al. 2004; Srivastava 2006). Therefore, we speculate that LYRM1 knockdown may break the balance between proliferation and apoptosis in P19 cells and then affect the differentiation of cardiac precursors into mature cardiomyocytes. However, the precise molecular mechanism that underlies this effect still remains unknown.

Apoptosis is the essential event of a vertebrate’s organ formation and development process. Too much or no degree of apoptosis can cause congenital heart defects (Fiorina et al. 2004). Michael et al. suggested that mitochondrial function influenced transmission and amplification of apoptosis signals (Crow et al. 2004). Cell apoptosis, in turn, induced dynamic changes in the function and structure of mitochondria (Martinou and Youle 2011; Ugarte-Uribe and Garcia-Saez 2014). Therefore, in the present study, we further explored the effects of LYRM1 knockdown on mitochondrial function in P19 cells.

We found that suppression of LYRM1 expression caused mitochondria dysfunction. Firstly, LYRM1 knockdown significantly increased the ROS level. Mitochondria are a major source of intracellular ROS, and mitochondria become dysfunctional and ROS accumulate as an intermediate with cell apoptosis increasing(Matsusaka and Ichikawa 1997). Then, the high concentrations of ROS further lead to mitochondrial dysfunction (Choksi et al. 2004). Furthermore, we observed that LYRM1 knockdown significantly decreased mitochondrial adenosine triphosphates (ATP) content and Mitochondrial Membrane Potential (MMP). It is well-known that the energy supply of cells is mainly generated from the mitochondrial ATP. We speculated that the decline in ATP synthesis accompanied by the impaired mitochondrial function would lead to an inadequate supply of cardiomyocyte energy, possibly inducing stagnation of cardiogenesis. In addition, MMP, an electrochemical gradient of protons between the inner and outer membranes, is fundamental for the conversion of ADP to ATP catalyzed by ATP synthase(Brown 1992), which can directly reflect mitochondrial function. The decreased MMP could cause mitochondria to swell, changing their permeability. Then, cytochrome c would release into the cytoplasm with the opening of the permeability transition (PT) pore and activate some protease systems, resulting in apoptosis increased (Tanaka et al. 2002). Therefore, we concluded that the increased apoptosis in LYRM1-silencing cells was likely to be associated with the increased ROS and the reduced MMP.

In theory, the deterioration of mitochondrial function would destroy the integrity of the mtDNA, resulting in a reduction of the mtDNA copy number. However, the difference in mtDNA copy number showed no statistical significance in fact. We speculated that there might be some other compensatory mechanisms that protected impaired mitochondria.

In conclusion, we demonstrated that LYRM1 knockdown significantly inhibited proliferation and differentiation and promoted apoptosis in P19 cells. In addition, LYRM1 knockdown induced mitochondrial impairment in P19 cells during differentiation, which was reflected in decreased MMP, cellular ATP production and increased ROS levels. These findings may provide a new viewpoint into the mechanisms of cardiac abnormalities. Furthermore, LYRM1 may be a potential new therapeutic target for CHDs. Therefore, the next step in this study would be to confirm whether abnormalities in LYRM1 knockdown contribute to CHD in vivo.
